# Supplementary material for: Ribosome inhibition by C9ORF72-ALS/FTD-associated poly-PR and poly-GR proteins revealed by cryo-EM
Source: Nat Commun. 2022 May 19;13:2776. doi: 10.1038/s41467-022-30418-0 (PMC9120013; doi:10.1038/s41467-022-30418-0)
Supplement: Supplementary file 1 — Supplementary Information [file 41467_2022_30418_MOESM1_ESM.pdf]

# Ribosome inhibition by *C9ORF72*-ALS/FTD-associated poly-PR and poly-GR proteins revealed by cryo-EM

Anna B. Loveland[1], Egor Svidritskiy[1], Denis Susorov[1], Soojin Lee[2], Alexander Park[1], Sarah Zvornicanin[1], Gabriel Demo[1,3], Fen-Biao Gao[2*] and Andrei A. Korostelev[1*]

Affiliations:

[1] RNA Therapeutics Institute, UMass Chan Medical School, 368 Plantation Street, Worcester, MA 01605, USA.

[2] Department of Neurology, UMass Chan Medical School, 368 Plantation Street, Worcester, MA 01605, USA.

[3] Central European Institute of Technology, Masaryk University, Kamenice 5, Brno, 625 00, Czech Republic

*Correspondence:Fen-Biao.Gao@umassmed.edu and Andrei.Korostelev@umassmed.edu

SUPPLEMENTARY INFORMATION: This file includes:

Table S1

Supplementary Fig. 1-7

Supplementary Note 1

Supplemental References

## Table S1: Cryo-EM data collection, refinement and validation statistics

| | S.c. 80S poly-GR | S.c. 80S poly-PR | O.c. 80S poly-PR | O.c. 80S poly-GR | E. c. 70S poly-PR |
|---|---|---|---|---|---|
| **Data collection and processing** | | | | | |
| Magnification | 47,619x | 47,619x | 57,471x | 60241x | 57,471x |
| Voltage (kV) | 300 | 300 | 200 | 300 | 200 |
| Electron exposure ($e^{-}/Å^2$) | 30 | 30 | 30 | 30 | 35 |
| Defocus range (μm) | 0.4-5 | 0.4-5 | 0.4-3 | 0.4-4 | 0.4-5 |
| Pixel size (Å) | 1.05 | 1.05 | 0.87 | 0.83 | 0.87 |
| Symmetry imposed | C1 | C1 | C1 | C1 | C1 |
| Initial particle images (no.) | 467,615 | 203,089 | 320,595 | 276,778 | 172,278 |
| Final particle images (no.) | 467,615 | 203,089 | 63,475 | 20,284 | 49,219 |
| Map resolution (Å)** | 2.7 | 2.4 | 3.1 | 2.9 | 2.9 |
| FSC threshold | 0.143 | 0.143 | 0.143 | 0.143 | 0.143 |
| Map resolution range (Å) | 2.5 - >8 | 2.2 - >8 | 2.8 - >8 | 2.6- >8 | 2.5 - >8 |
| **Refinement** | | | | | |
| Initial model used (PDB code) | 4V88 | 4V88 | 6R6P | 6R5Q | 5UYM |
| Model resolution (Å)* | 2.7 | 2.4 | 3.1 | 2.9 | 2.9 |
| FSC threshold | 0.143 | 0.143 | 0.143 | 0.143 | 0.143 |
| Model resolution range (Å) | 2.5 - >8 | 2.2 - >8 | 2.8 - >8 | 2.7- >8 | 2.5 - >8 |
| Correlation Coefficient (cc_mask)* | 0.89 | 0.89 | 0.84 | 0.83 | 0.83 |
| Real-space R-factor † | 0.21 | 0.24 | 0.22 | 0.22 | 0.23 |
| Map-sharpening *B* factor ($Å^2$) | -25 | -32 | 0 | 0 | -35 |
| Model composition* | | | | | |
| Non-hydrogen atoms | 125,396 | 125,434 | 213,639 | 213,256 | 145,427 |
| Protein residues | 6,239 | 6,241 | 11,381 | 11,391 | 6,104 |
| RNA residues | 3,550 | 3,550 | 5,702 | 5,680 | 4,555 |
| *B* factors ($Å^2$)* | | | | | |
| Protein | 64.4 | 50.6 | 191.6 | 141.0 | 138.6 |
| RNA | 76.0 | 58.3 | 176.0 | 157.2 | 119.9 |
| R.m.s. deviations*,§ | | | | | |
| Bond lengths (Å) | 0.002 | 0.002 | 0.003 | 0.003 | 0.003 |
| Bond angles (°) | 0.5 | 0.5 | 0.8 | 0.8 | 0.7 |
| Validation* | | | | | |
| MolProbity score | 1.44 | 1.42 | 1.82 | 1.72 | 1.71 |
| Clashscore | 3.43 | 3.65 | 9.92 | 9.46 | 7.87 |
| Poor rotamers (%) | 0.02 | 0.0 | 0.05 | 0.02 | 0.06 |
| Ramachandran plot* | | | | | |
| Favored (%) | 95.6 | 96.1 | 95.7 | 96.6 | 95.9 |
| Allowed (%) | 4.4 | 3.9 | 4.3 | 3.4 | 4.1 |
| Disallowed (%) | 0.0 | 0.0 | 0.03 | 0.04 | 0.0 |
| Validation (RNA)* | | | | | |
| Good sugar pucker (%) | 99.1 | 99.2 | 98.6 | 98.7 | 99.6 |
| Good backbone (%)# | 85.4 | 85.7 | 78.5 | 79.6 | 83.2 |

**** from Frealign (FSC_part)**
*** from Phenix**
**† from RSRef**
**§ root mean square deviations**
**# RNA backbone suites that fall into recognized rotamer conformations defined by Molprobity**

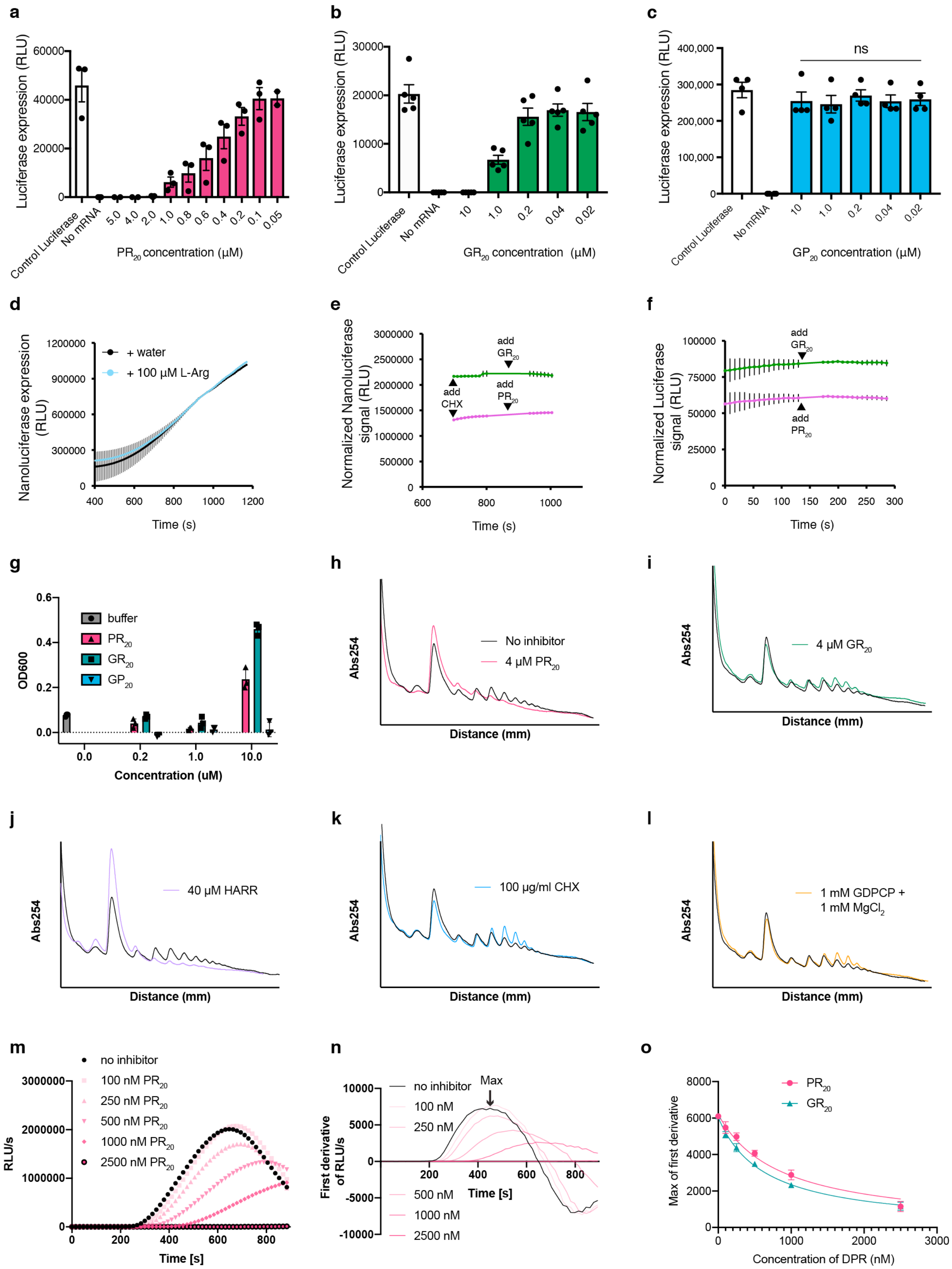

a
Luciferase expression (RLU)
60000
40000
20000
0
Control Luciferase
No mRNA
5.0
4.0
2.0
1.0
0.8
0.6
0.4
0.2
0.1
0.05
PR20 concentration (µM)
b
30000
20000
10000
0
Luciferase expression (RLU)
Control Luciferase
No mRNA
10
1.0
0.2
0.04
0.02
GR20 concentration (µM)
c
400,000
300,000
200,000
100,000
0
Luciferase expression (RLU)
ns
Control Luciferase
No mRNA
10
1.0
0.2
0.04
0.02
GP20 concentration (µM)
d
Nanoluciferase expression (RLU)
1200000
900000
600000
300000
0
+ water
+ 100 µM L-Arg
400
600
800
1000
1200
Time (s)
e
Normalized Nanoluciferase signal (RLU)
3000000
2500000
2000000
1500000
1000000
500000
0
add GR20
add CHX
add PR20
600
800
1000
Time (s)
f
Normalized Luciferase signal (RLU)
100000
75000
50000
25000
0
add GR20
add PR20
0
50
100
150
200
250
300
Time (s)
g
OD600
0.6
0.4
0.2
0.0
buffer
PR20
GR20
GP20
0.0
0.2
1.0
10.0
Concentration (uM)
h
Abs254
No inhibitor
4 µM PR20
Distance (mm)
i
Abs254
4 µM GR20
Distance (mm)
j
Abs254
40 µM HARR
Distance (mm)
k
Abs254
100 µg/ml CHX
Distance (mm)
l
Abs254
1 mM GDPCP +
1 mM MgCl2
Distance (mm)
m
RLU/s
3000000
2000000
1000000
0
no inhibitor
100 nM PR20
250 nM PR20
500 nM PR20
1000 nM PR20
2500 nM PR20
0
200
400
600
800
Time [s]
n
First derivative of RLU/s
10000
5000
0
-5000
-10000
Max
no inhibitor
100 nM
250 nM
500 nM
1000 nM
2500 nM
200
400
600
800
Time [s]
o
Max of first derivative
8000
6000
4000
2000
0
PR20
GR20
0
1000
2000
3000
Concentration of DPR (nM)

**Supplementary Fig. 1. Control experiments for inhibition of translation by DPRs in RRL.** (**a-c**) $PR_{20}$ and $GR_{20}$ but not $GP_{20}$ inhibit translation of firefly luciferase mRNA in RRL. Relative luminescence units (RLU) after 1 hour of translation in the absence (positive luciferase control and negative “no mRNA” control) or presence of DPRs are shown; n=3, 5 and 4, respectively, with independent experiments and error bars representing mean ± SEM. Non-significance indicated in panel (**c**) was determined using a one-way ANOVA with Dunnett’s post-hoc test for multiple comparisons. (**d**) 100 μM L-Arg does not inhibit the translation of nanoluciferase in RRL. n=3 independent experiments; mean ± SD. (**e-f**) $PR_{20}$ and $GR_{20}$ do not affect enzyme activity of nanoluciferase (**e**) and firefly luciferase (**f**). (**e**) Nanoluciferase mRNA was translated in the absence of DPRs, translation was stopped by cycloheximide (CHX), then 1 μM $PR_{20}$ or $GR_{20}$ were added and luminescence was monitored. n=8 independent experiments; mean of normalized RLU ± SD. (**f**) 40 nM firefly luciferase enzyme (Promega) was supplemented into RRL in the absence of mRNA and amino acids, and after 120 seconds 1 μM $PR_{20}$ or $GR_{20}$ were added. n=6 independent experiments; mean of normalized RLU ± SD. (**g**) Concentrations of $PR_{20}$ and $GR_{20}$ that inhibit RRL translation and peptidyl transfer (from 0.2 to 1 μM) do not cause HEK293 RNA aggregation nor does water or $GP_{20}$, measured as 600 nm light absorbance. 10 μM $PR_{20}$ and $GR_{20}$ did not induce visible opalescence but result in increased OD600, consistent with some RNA aggregation. n=3 independent experiments; mean ± SD. (**h-l**) Comparison of polysome profiles of rabbit reticulocyte lysate translating endogenous mRNA in the presence of translation inhibitors. The translation mixture was incubated for 5 minutes at 37°C in the presence or absence of $PR_{20}$, $GR_{20}$, harringtonine, cycloheximide and GDPCP (n=2

independent experiments; representative traces with concurrent uninhibited control are shown). Under these conditions, $PR_{20}$ behaves more like harringtonine stalling the initiating 80S ribosome and allowing polysome run off. (**m**) One of the replicates of nanoluciferase translation assay and its inhibition by $PR_{20}$. Nanoluciferase signal (RLU/s) was followed over 900 s. (**n**) First derivative of nanolucifase signal over time was calculated and the maximum rate was taken for $K_i$ fitting as in panel **o**. (**o**) To obtain the IC50 for translation inhibition by $GR_{20}$ and $PR_{20}$, the mean of three maximal rates (n=3 independent experiments) similar to (**m-n**) was plotted versus inhibitor concentration and was fitted to a 3-parameter inhibition model (Y=baseline+(max-baseline)/(1+X/IC50)) to obtain the IC50 values where the baseline was constrained to 0. Source data are provided as a Source Data file.

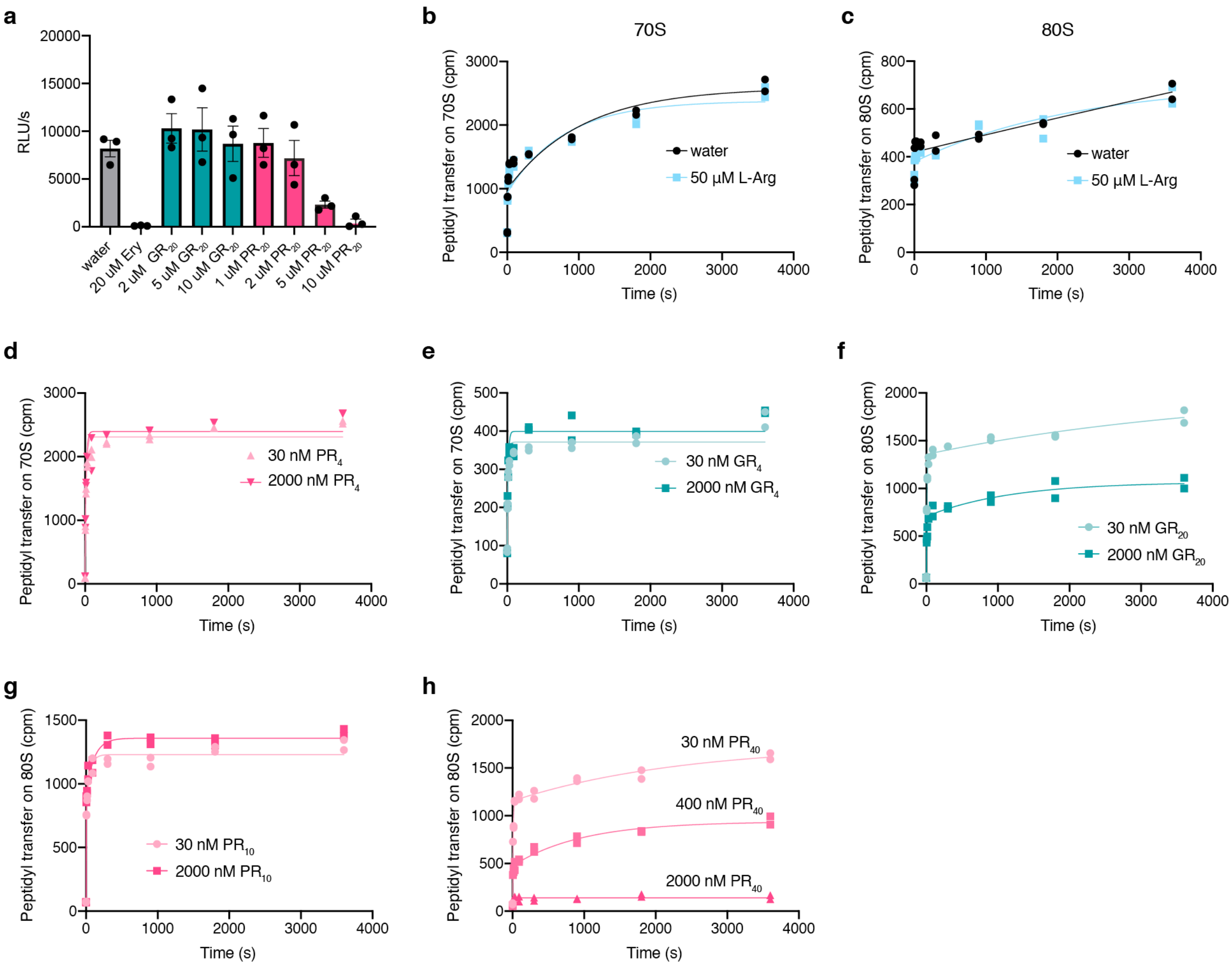

**Supplementary Fig. 2: Control experiments for inhibition of peptidyl transfer by DPRs** (**a**) Inhibition of nanoluciferase translation in *E. coli* cell extracts (NEBExpress) is seen by the antibiotic erythromycin (Ery) and ≥5 μM $PR_{20}$, but not by $GR_{20}$ (n=3 independent experiments, mean of slope from 100-200s ± SEM). (**b-c**) Time progress curves of the puromycin reaction showing that 50 μM L-Arg (blue squares) does not inhibit peptidyl transfer on *E. coli* 70S ribosomes (**b**) and rabbit 80S ribosomes (**c**). (**d-e**) Time progress curves of the puromycin reaction on *E. coli* 70S ribosomes showing that $PR_4$ and $GR_4$ do not inhibit peptidyl transfer at high (2 μM) concentration. (**f**) Time progress curves of the puromycin reaction on rabbit 80S ribosomes showing that $GR_{20}$ inhibits peptidyl transfer at 2 μM. (**g**) Time progress curves of the puromycin reaction on

rabbit 80S ribosomes showing $PR_{10}$ does not inhibit peptidyl transfer at 2 μM. (**h**) Time progress curves of the puromycin reaction on rabbit 80S ribosomes showing $PR_{40}$ strongly inhibits peptidyl transfer (n=2 in panels **b-h**). Source data are provided as a Source Data file.

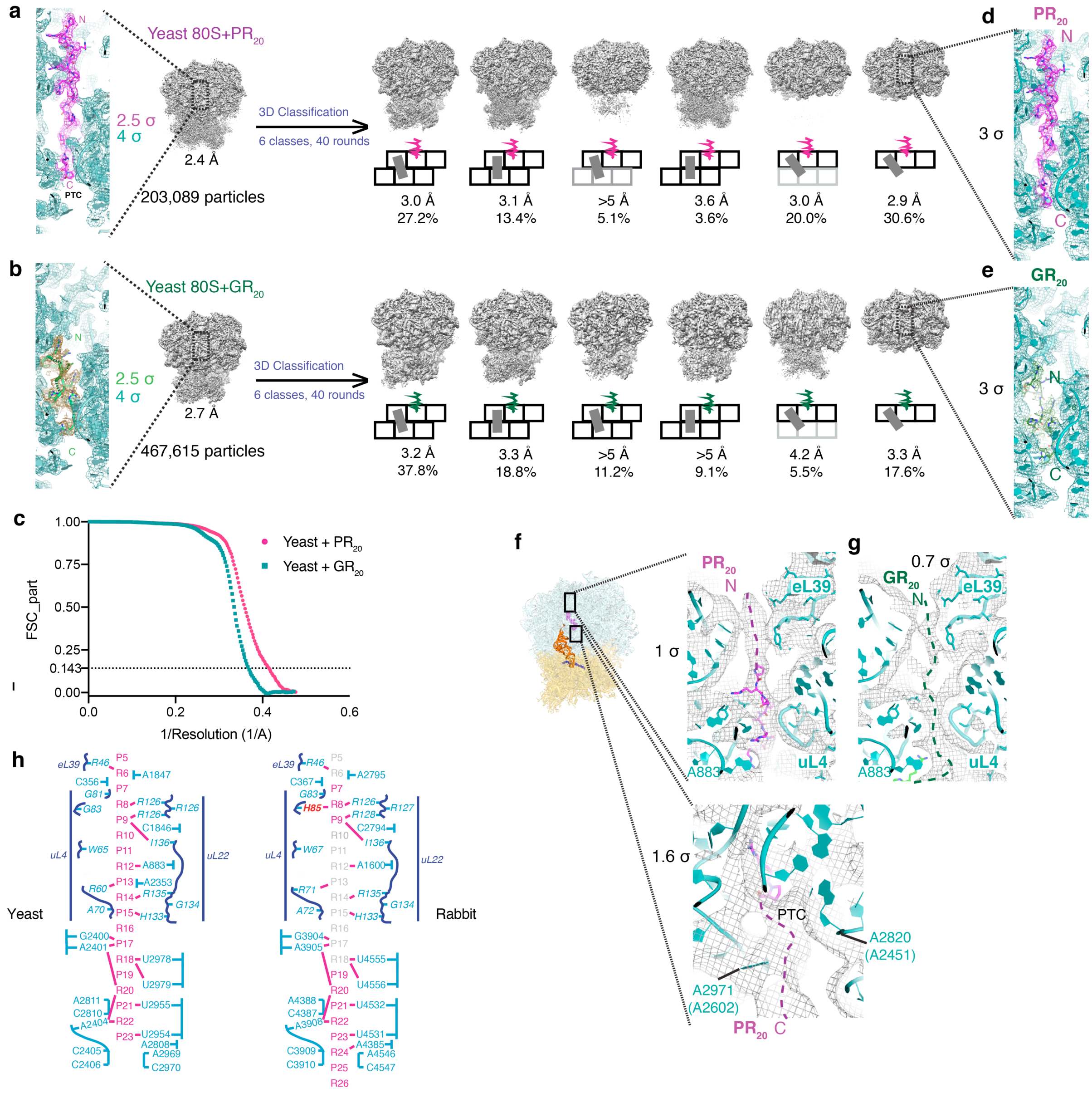

**Supplementary Fig. 3: Cryo-EM structures of yeast 80S ribosomes with $PR_{20}$ or $GR_{20}$.** (**a**) Maximum likelihood classification for the yeast 80S•$tRNA^{fMet}$•$PR_{20}$ dataset shows $PR_{20}$ binding at the polypeptide tunnels of 80S and 60S particles. Cartoons depict the states/conformations of the ribosome as follows: black grid are large (top) and small (bottom) subunits with their respective E (left square), P (middle square), and A (right square) sites. Gray bars are tRNA bound to these sites. Decreased width of tRNA or gray grid indicates partial occupancy. Offset of top and bottom rows indicate rotation of the small subunit relative to the large subunit. Magenta squiggle indicates $PR_{20}$ bound to the polypeptide tunnel. On the left, the cryo-EM map, without B-factor sharpening, is shown at 2.5 σ (for $PR_{20}$) and 4 σ (for the ribosome). (**b**) Maximum likelihood classification for the yeast 80S•$tRNA^{fMet}$•$GR_{20}$ dataset shows $GR_{20}$ binding at the polypeptide tunnels of 80S and 60S particles. Cartoon schematics are as in (**a**), except that green squiggle indicates $GR_{20}$ bound to the polypeptide tunnel. On the left, the cryo-EM map, without B-factor sharpening, is shown at 2.5 σ (for $GR_{20}$) and 4 σ (for the ribosome). (**c**) Fourier shell correlation (FSC) curves for cryo-EM maps used for model building and structure refinements. (**d**) Cryo-EM density for $PR_{20}$ bound to the yeast 60S ribosomal subunit. Cryo-EM map is shown at 3 σ. (**e**) Cryo-EM density for $GR_{20}$ bound to the yeast 60S ribosomal subunit. Cryo-EM map is shown at 3 σ. (**f**) The yeast 80S ribosome with $PR_{20}$ contains lower-resolution features extending away from the modeled density towards 60S surface (top panel) and from the peptidyl transferase center (PTC) to intersubunit space (bottom panel). The cryo-EM map was low-pass filtered to 5 Å, and a B-factor of 100 $Å^2$ was applied and the mesh is shown at 1 σ and 1.6 σ for the top and bottom panel, respectively. (**g**) The yeast 80S ribosome with $GR_{20}$ contains lower-

resolution features extending away from the modeled density towards 60S surface. The cryo-EM map was low-pass filtered to 5 Å, and a B-factor of 100 $Å^2$ was applied and is shown at 0.7 σ. (**h**) The ribosomal residues of the polypeptide tunnel (blue) that interact with $PR_{20}$ (magenta) are conserved between yeast (left) and rabbit (right) with one exception (red, a glycine residue in yeast uL4 is a histidine in rabbit uL4). Protein residues are shown in italics, rRNA are regular print. Nucleobase and side chain interactions are shown by pink dashes, backbone interactions are shown by proximity. Source data are provided as a Source Data file.

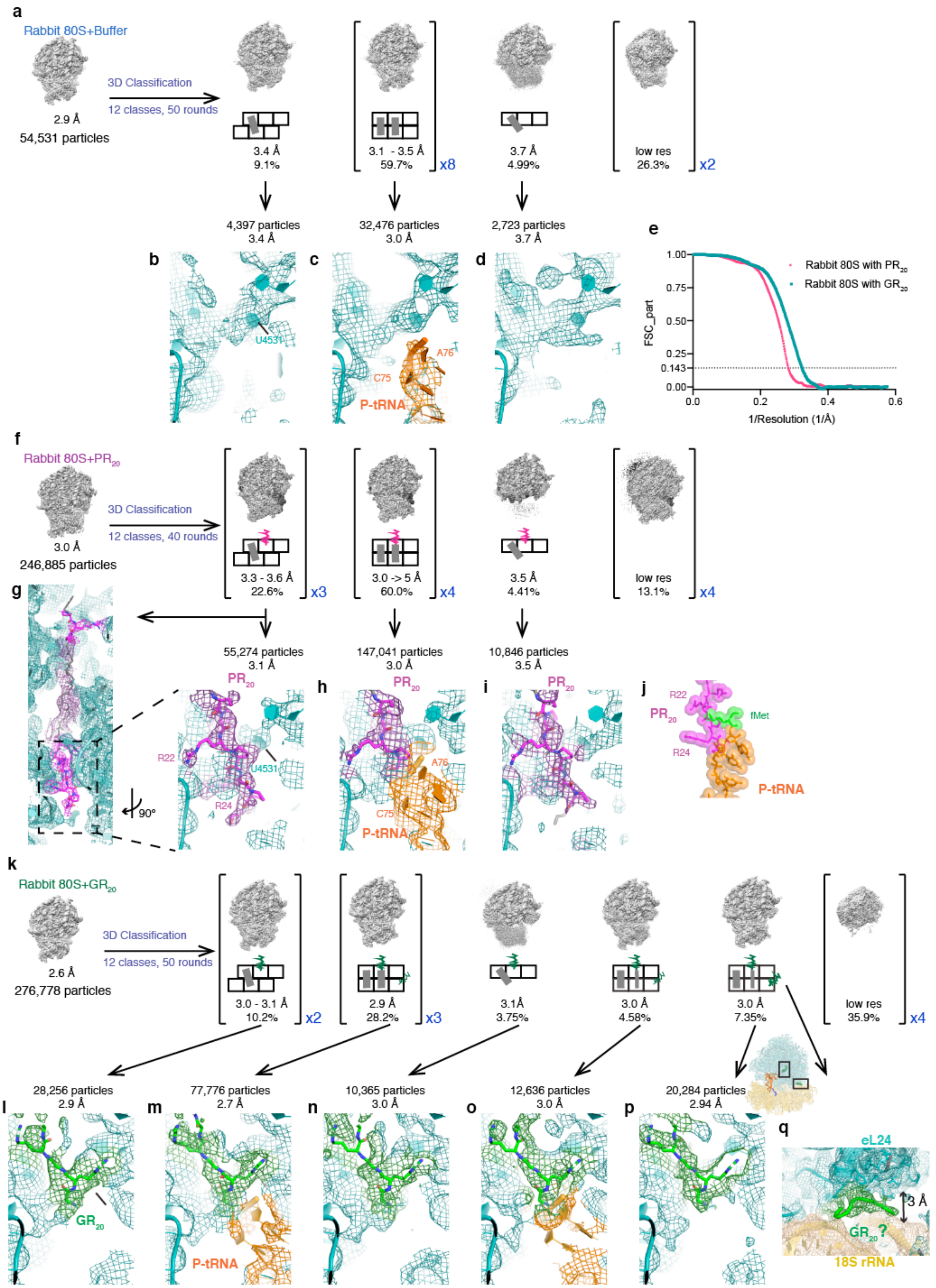

a
Rabbit 80S+Buffer
3D Classification
12 classes, 50 rounds
2.9 Å
54,531 particles
3.4 Å
9.1%
3.1 - 3.5 Å
59.7%
x8
3.7 Å
4.99%
low res
26.3%
x2
4,397 particles
3.4 Å
32,476 particles
3.0 Å
2,723 particles
3.7 Å
b
U4531
c
A76
C75
P-tRNA
d
e
Rabbit 80S with PR20
Rabbit 80S with GR20
FSC_part
1/Resolution (1/Å)
f
Rabbit 80S+PR20
3D Classification
12 classes, 40 rounds
3.0 Å
246,885 particles
3.3 - 3.6 Å
22.6%
x3
3.0 -> 5 Å
60.0%
x4
3.5 Å
4.41%
low res
13.1%
x4
g
55,274 particles
3.1 Å
147,041 particles
3.0 Å
10,846 particles
3.5 Å
PR20
R22
U4531
R24
90°
h
A76
C75
P-tRNA
i
j
R22
PR20
fMet
R24
P-tRNA
k
Rabbit 80S+GR20
3D Classification
12 classes, 50 rounds
2.6 Å
276,778 particles
3.0 - 3.1 Å
10.2%
x2
2.9 Å
28.2%
x3
3.1Å
3.75%
3.0 Å
4.58%
3.0 Å
7.35%
low res
35.9%
x4
28,256 particles
2.9 Å
77,776 particles
2.7 Å
10,365 particles
3.0 Å
12,636 particles
3.0 Å
20,284 particles
2.94 Å
l
GR20
m
P-tRNA
n
o
p
q
eL24
3 Å
GR20 ?
18S rRNA

**Supplementary Fig. 4: Cryo-EM structures of rabbit 80S ribosomes with buffer (negative control), $GR_{20}$ or $PR_{20}$.**

(**a**) Maximum likelihood classification of the dataset for rabbit 80S•$tRNA^{fMet}$ in the absence of DPRs (Buffer control) reveals 80S maps with different intersubunit rotation and tRNA occupancy, and 60S maps. (**b-d**) The PTC is either empty (**b,d**) or occupied only by P-tRNA (**c**). Map (mesh) is shown at 4 σ. The structural model was a rigid-body fit of the 60S subunit (PDB: 6R5Q; https://www.rcsb.org/structure/6R5Q)[1] to each map and P-site $tRNA^{fMet}$ is from (PDB:5UYM; https://www.rcsb.org/structure/5UYM)[2]. (**e**) Fourier shell correlation (FSC) curves for cryo-EM maps used for model building and structure refinements. (**f**) Maximum likelihood classification of the rabbit 80S•$tRNA^{fMet}$ + $PR_{20}$ dataset reveals 80S maps with different small-subunit rotation, tRNA occupancy, $PR_{20}$ features and 60S maps. (**g**) Density for $PR_{20}$ in the cryo-EM map for the hybrid-state (rotated) 80S particles with P/E tRNA. Right panel: map is shown contoured at 4σ (cyan) around ribosomal residues and at 2.5σ (magenta) around PR residues within the PTC and near uL22. Continuous lower-resolution density between these regions is shown using a low-pass-filtered map (to 4 Å, B-factor softened by applying a B-factor of 50) at 1.1σ (dark purple). Left panel: close up in the PTC is shown at a single contour (3σ). (**h**) Densities for $PR_{20}$ and P-site tRNA are in close contact in the cryo-EM map of the classical (non-rotated) rabbit 80S ribosome bound with P-site tRNA. $PR_{20}$ appears displaced down the tunnel, compared to that in the absence of P-site tRNA. (**i**) Density for $PR_{20}$ in the cryo-EM map of rabbit 60S particles is similar to that in the 80S particles (see **g**). Map (mesh) is shown at 3 σ in panels **g-i**. Model shown was refined into map shown in (**g**) and then rigid-body fit into maps shown in **h** and **i**. (**j**) An aminoacyl or

peptidyl moiety at A76 of the P-site tRNA would sterically clash with $PR_{20}$. (**k**) Maximum likelihood classification for the rabbit 80S•$tRNA^{fMet}$ + $GR_{20}$ dataset reveals 80S maps with different small-subunit rotation, tRNA occupancy, $GR_{20}$ features and 60S maps. (**l**) Density for $GR_{20}$ in the cryo-EM map for the hybrid-state (rotated) 80S particles with P/E tRNA. (**m**) Densities for $GR_{20}$ and P-site tRNA are in close contact in the cryo-EM map of the classical (non-rotated) rabbit 80S ribosome bound with P-site tRNA. (**n**) Density for $GR_{20}$ in the cryo-EM map of rabbit 60S particles is similar to that in the 80S particles. (**o**) Density for $GR_{20}$ is seen while the CCA of P-tRNA is very weak in a cryo-EM map that shows a classical (non-rotated) ribosome conformation and weak density for P-tRNA in 60S subunit. (**p-q**) Density for $GR_{20}$ is seen at the PTC (**p**) and putative density is observed at a second site (**q**) at the subunit interface near eL24, which may correspond to poly-GR or a previously unmodeled conformation of eL24's C-terminal domain (but putative GR backbone is shown) in a cryo-EM map showing a non-rotated conformation with an extra 2.8-Å widening of the subunit interface. (**l-q**) Map is shown at 4 σ (**l-p**) or 3 (**q**). Model shown in (**p-q**) was built and refined into map in (**p-q**) and was rigid-body fit into maps in (**l-o**) along with $tRNA^{fMet}$ from (PDB:5UYM; https://www.rcsb.org/structure/5UYM)[2] in (**m,o**). The CCA end of P-tRNA is disordered in this map and was not modeled. Source data are provided as a Source Data file.

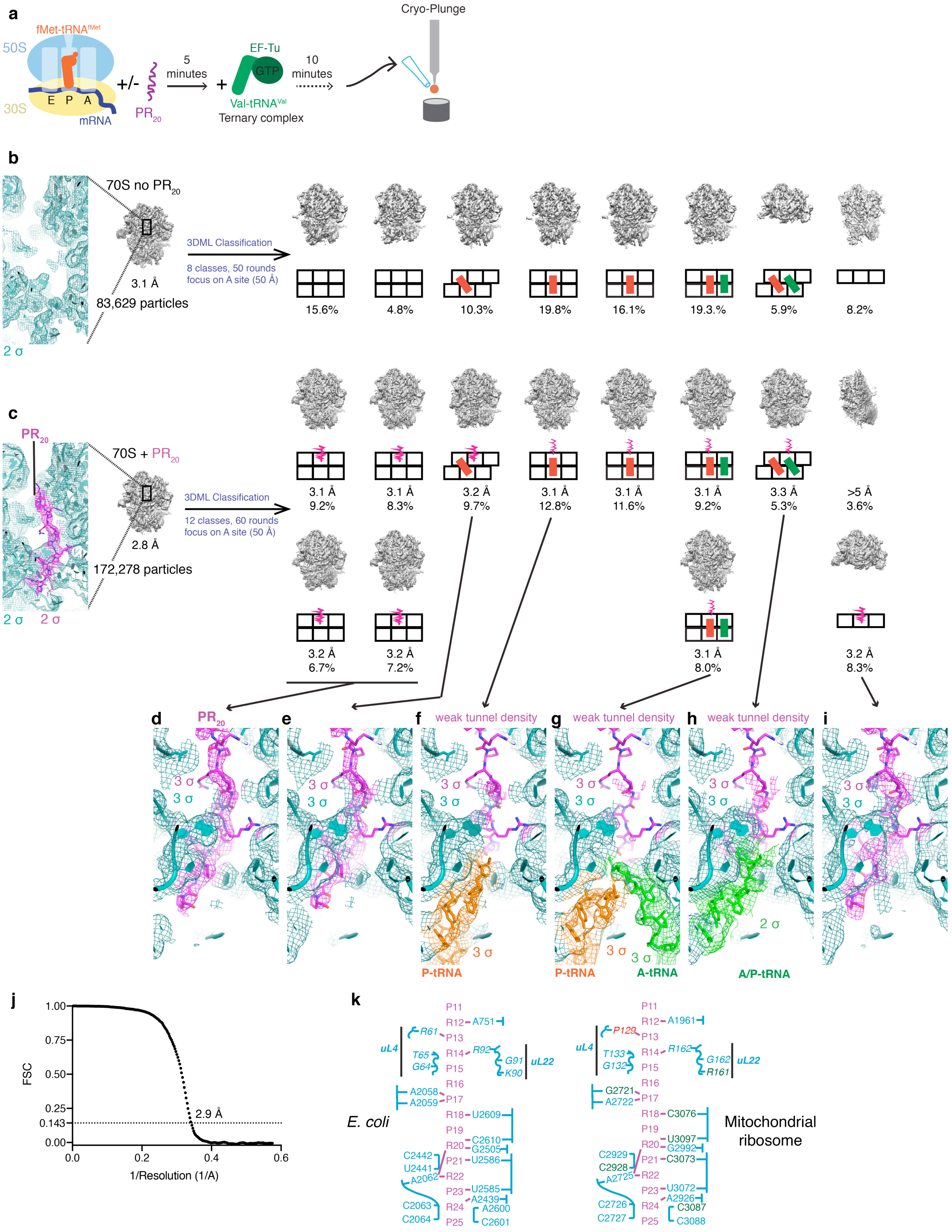

a
fMet-tRNAfMet
50S
30S
E P A
mRNA
+/-
PR20
5 minutes
EF-Tu
GTP
Val-tRNAVal
Ternary complex
10 minutes
Cryo-Plunge
b
70S no PR20
3DML Classification
8 classes, 50 rounds focus on A site (50 Å)
3.1 Å
83,629 particles
2 σ
15.6%
4.8%
10.3%
19.8%
16.1%
19.3.%
5.9%
8.2%
c
PR20
70S + PR20
3DML Classification
12 classes, 60 rounds focus on A site (50 Å)
2.8 Å
172,278 particles
2 σ
2 σ
3.1 Å 9.2%
3.1 Å 8.3%
3.2 Å 9.7%
3.1 Å 12.8%
3.1 Å 11.6%
3.1 Å 9.2%
3.3 Å 5.3%
>5 Å 3.6%
3.2 Å 6.7%
3.2 Å 7.2%
3.1 Å 8.0%
3.2 Å 8.3%
d
PR20
3 σ
3 σ
e
3 σ
3 σ
f
weak tunnel density
3 σ
3 σ
3 σ
P-tRNA
g
weak tunnel density
3 σ
3 σ
3 σ
3 σ
P-tRNA
A-tRNA
h
weak tunnel density
3 σ
3 σ
2 σ
A/P-tRNA
i
3 σ
3 σ
j
FSC
1.00
0.75
0.50
0.25
0.143
0.00
0.0
0.2
0.4
0.6
2.9 Å
1/Resolution (1/A)
k
E. coli
uL4
R61
T65
G64
P11
R12
A751
P13
R14
R92
P15
G91
K90
uL22
R16
A2058
A2059
P17
R18
U2609
P19
R20
C2610
G2505
C2442
U2441
A2062
P21
U2586
R22
P23
U2585
A2439
R24
A2600
C2063
C2064
P25
C2601
Mitochondrial ribosome
uL4
P129
T133
G132
P11
R12
A1961
P13
R14
R162
P15
G162
R161
uL22
R16
G2721
A2722
P17
R18
C3076
P19
R20
U3097
G2992
C2929
C2928
A2725
P21
C3073
R22
P23
U3072
A2926
R24
C3087
C2726
C2727
P25
C3088

**Supplementary Fig. 5**: **Cryo-EM analyses of *E. coli* 70S ribosomes during elongation in the presence of $PR_{20}$.** (**a**) Scheme for a translation elongation reaction on the 70S ribosome with or without $PR_{20}$, captured by cryo-EM. (**b**) Schematics of the maximum likelihood classification of the dataset for 70S with the Buffer control**.** (**c**) Maximum likelihood classification of the 70S-$PR_{20}$ dataset. (**d)** $PR_{20}$ occupies the polypeptide tunnel of the 70S ribosome. $PR_{20}$ was built into the 70S map containing no tRNA (2.9 Å resolution). The map was B-factor sharpened (-32 $Å^2$) and is shown at 3 σ. (**e**) $PR_{20}$ occupies the polypeptide tunnel of the 70S ribosome in the hybrid state with P/E tRNA. Model of the 50S and $PR_{20}$ from (**d**) were rigid-body fit into map of the 70S ribosome with rotated 30S subunit bound with P/E tRNA. Map is shown, without sharpening, at 3 σ. (**f**) Density in the tunnel is weak in the presence of fMet-tRNA (orange). 50S and $PR_{20}$ from d) were rigid-body fit into the map of 70S with P- and E-site tRNAs. P-site tRNA (fMet-$tRNA^{fMet}$) from PDB: 5UYM, https://www.rcsb.org/structure/5UYM [2] is shown. (**g**) Density in the tunnel is weak in the presence of both the A-site (green) and P-site (orange) tRNAs. Deacylated P-tRNA (P site) and dipeptidyl-tRNA (A site) from PDB: IVY5, https://www.rcsb.org/structure/IVY5 [3] are shown. (**h**) Density in the tunnel is weak in the presence of the product dipeptidyl-tRNA (A/P hybrid state; green). Analog of dipeptidyl-A/P-tRNA from PDB:1M90, https://www.rcsb.org/structure/1M90 [4] is shown. (**i**) Strong density for $PR_{20}$ in the polypeptide tunnel of the 50S ribosomal subunit. (**j**) Fourier shell correlation (FSC) curve for the 70S map used to model the 70S•$PR_{20}$ structure (see panel **c**). (**k**) Comparison of the *E. coli* polypeptide tunnel with that of the human mitochondrial ribosome. PR residues from the *E. coli* cryo-EM structure (this work) are shown (pink). Interacting

ribosomal protein residues are shown in italics, rRNA are regular print (blue): nucleobase and side chain interactions are shown by pink dashes, backbone interactions are shown by proximity. Conservative differences (ex. purine/purine) are green, non-conservative difference R61/P129 is red. Source data are provided as a Source Data file.

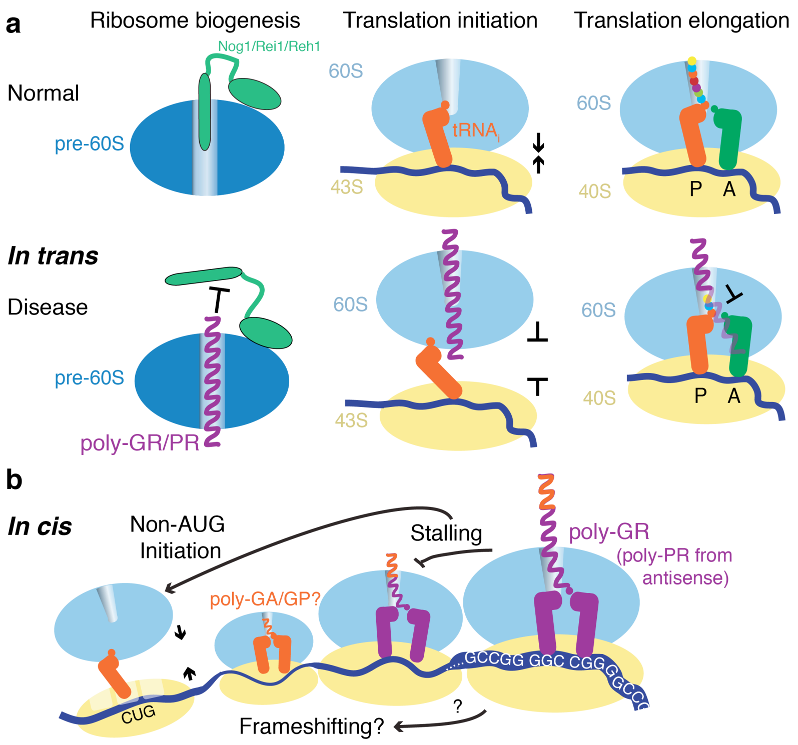


**Supplementary Fig. 6: Proposed mechanisms of translation repression caused by poly-PR and poly-GR binding to the ribosomal polypeptide tunnel.** (**a**) *In trans* binding of poly-PR and poly-GR (magenta) to 60S (blue) polypeptide tunnel is consistent with perturbation of 60S biogenesis [5-7] and translation initiation [8], while binding to 80S inhibits translation elongation (this work and [9]). Similarly, binding to large mitochondrial subunits and mitochondrial ribosomes may perturb mitochondrial function [10,11]. Biogenesis factors Nog1/Rei1/Reh1 are green, 40S subunit yellow, tRNAs in the P and A sites are orange and green. (**b**) Translation of $G_4C_2$ repeats may cause stalling of poly-PR and poly-GR in the ribosome, resulting in translation inhibition *in cis*. If transient, the stalling may enhance otherwise inefficient non-AUG initiation and/or frameshifting [12] of the $G_4C_2$ repeat sequence [13], leading to amplified translation of the DPR proteins (tRNA/peptide is orange for non-arginine DPR proteins and magenta for arginine-containing DPR proteins).

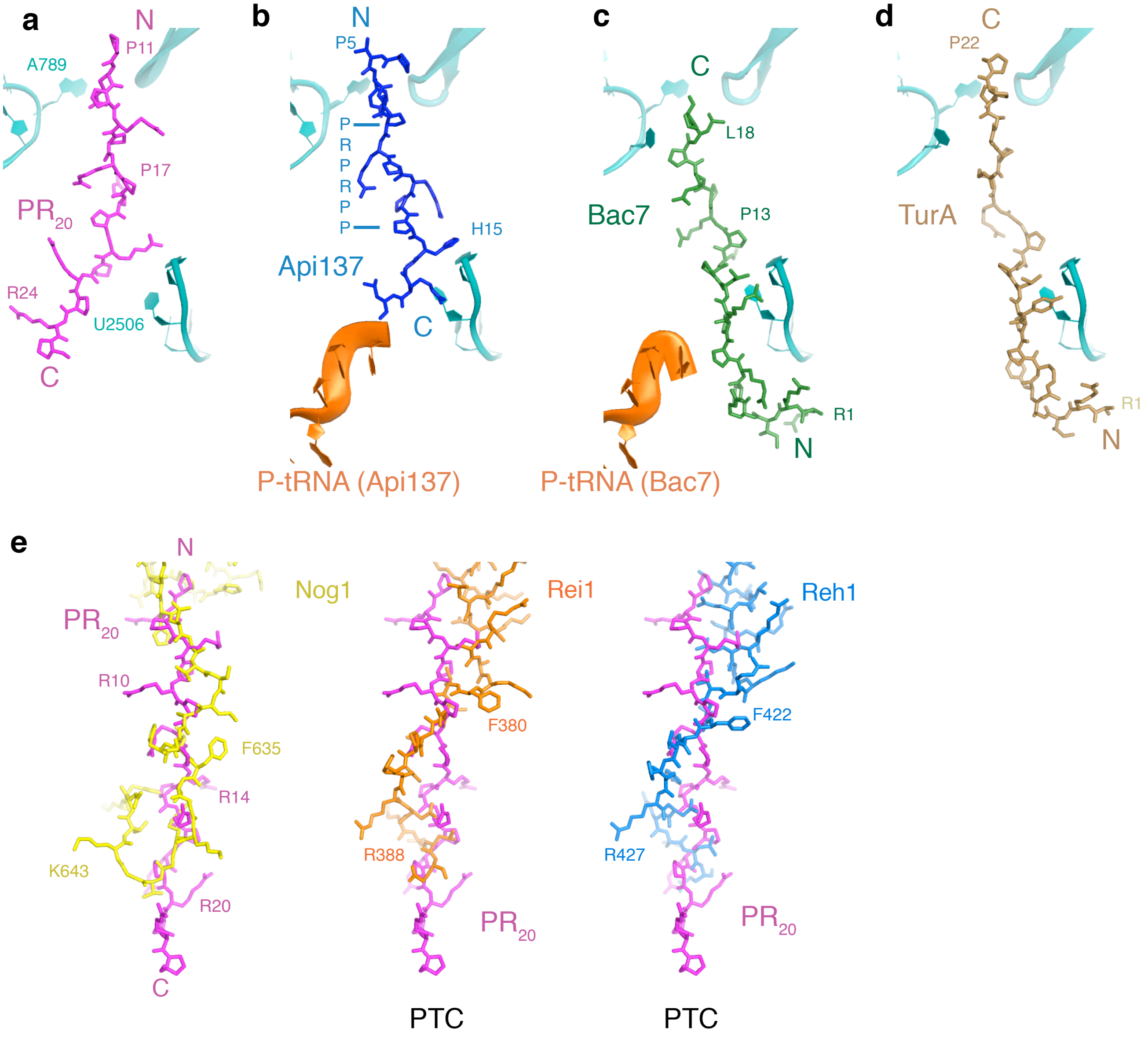


**Supplementary Fig. 7**: **Binding sites of $PR_{20}$ and $GR_{20}$ overlap with those of eukaryotic proline-rich antimicrobial peptides (PrAMPs) and 60S ribosomal subunit biogenesis factors**. (**a-d**) Comparison of the position of $PR_{20}$ in the 70S tunnel (**a**, magenta; this work) with those of eukaryotic proline-rich antimicrobial peptides (PrAMPs) bound to bacterial 70S ribosomes: (**b**) Api137 (blue; PDB:5O2R, https://www.rcsb.org/structure/5O2R [14]) (**c**) Bac7 (green; PDB:5HAU, https://www.rcsb.org/structure/5HAU [15]), (**d**) TurA (brown; PDB: 6FKR,

https://www.rcsb.org/structure/6FKR [16]. Ribosomal protein uL22 and helices 35a and 90 of 23S rRNA of the polypeptide tunnel are shown in cyan for reference. (**e**) Cryo-EM structures of yeast 60S biogenesis intermediates bound with Nog1 (yellow), Rei1 (orange) and Reh1 (blue) (PDB: 3JCT, https://www.rcsb.org/structure/3JCT [17], PDB:5APN, https://www.rcsb.org/structure/5APN [18] and PDB:5H4P, https://www.rcsb.org/structure/5H4P [19]) show that the binding sites overlap with that of $PR_{20}$ in the yeast 60S subunit (magenta; this work).

**Supplementary Note 1**

*Probable interference of poly-PR and poly-GR with ribosome biogenesis, mitochondrial translation and $G_4C_2$-repeat translation.*

Our findings suggest how poly-PR and poly-GR could interfere with other cellular processes involving the ribosome, which are beyond the scope of this study but supported by published research. First, consistent with cellular co-localization of the arginine-containing DPR proteins with nucleoli in cell lines and patient tissues [5-7,20,21], impaired ribosomal rRNA biogenesis in cells expressing DPR proteins [5,21], and the identification of ribosomal biogenesis factors as DPR interactors [9,20-22], DPR binding to the maturing pre-60S particles might disrupt ribosome biogenesis. Indeed, the DPR protein binding site directly overlaps with ribosome biogenesis factors Nog1, Rei1, and Reh1, which enter the ribosomal polypeptide tunnel to test the integrity of the maturing 60S subunit (**Supplementary Fig. 6a, Supplementary Fig. 7e**) [17-19].

Next, the structural and functional conservation suggests that arginine-containing DPRs could bind and inhibit mitochondrial ribosomes (**Supplementary Fig. 5k)**, consistent with the association of cellular DPR proteins with mitochondrial ribosome components [7,11,22] and mitochondrial dysfunction associated with *C9ORF72* repeat expansions [10,11].

Finally, poly-GR and/or poly-PR could affect translation of extended $G_4C_2$- or $G_2C_4$-repeat RNA. Here, nascent poly-GR and poly-PR could bind tightly to the polypeptide tunnel and stall ribosomes *in cis* (**Supplementary Fig. 6b**). Indeed, when we finalized this work, a study reported that poly-PR and poly-GR but not poly-GA or poly-AP stall translation *in cis*, requiring more than 10 repeats for efficient stalling [22]. Co-translational stalling may result in accumulation of DPR-stalled polysomes [7,23], consistent with DPR-

and ribosome-containing aggregates in patient cells [7,24], and trigger cellular stress [21]. Increased cellular stress may enhance DPR protein synthesis [25,26], eliciting a positive feedback loop between DPR protein accumulation and cellular stress. Indeed, translational stalling was recently shown to enhance translation from non-AUG codons [12]. Since translation of $G_4C_2$ mRNA repeats depends on non-AUG initiation and possibly frameshifting [13], translation of arginine-containing DPR proteins may increase cellular production of these and/or other DPR proteins [23], such as poly-GA and poly-GP.

**Supplemental References**


1. Shanmuganathan, V. *et al.* Structural and mutational analysis of the ribosome-arresting human XBP1u. *Elife* **8**, doi:10.7554/eLife.46267 (2019).
2. Loveland, A. B., Demo, G., Grigorieff, N. & Korostelev, A. A. Ensemble cryo-EM elucidates the mechanism of translation fidelity. *Nature* **546**, 113-117, doi:10.1038/nature22397 (2017).
3. Polikanov, Y. S., Steitz, T. A. & Innis, C. A. A proton wire to couple aminoacyl-tRNA accommodation and peptide-bond formation on the ribosome. *Nat Struct Mol Biol* **21**, 787-793, doi:10.1038/nsmb.2871 (2014).
4. Hansen, J. L., Schmeing, T. M., Moore, P. B. & Steitz, T. A. Structural insights into peptide bond formation. *Proc Natl Acad Sci U S A* **99**, 11670-11675 (2002).
5. Kwon, I. *et al.* Poly-dipeptides encoded by the C9orf72 repeats bind nucleoli, impede RNA biogenesis, and kill cells. *Science* **345**, 1139-1145, doi:10.1126/science.1254917 (2014).
6. Wen, X. *et al.* Antisense proline-arginine RAN dipeptides linked to C9ORF72-ALS/FTD form toxic nuclear aggregates that initiate in vitro and in vivo neuronal death. *Neuron* **84**, 1213-1225, doi:10.1016/j.neuron.2014.12.010 (2014).
7. Hartmann, H. *et al.* Proteomics and C9orf72 neuropathology identify ribosomes as poly-GR/PR interactors driving toxicity. *Life Sci Alliance* **1**, e201800070, doi:10.26508/lsa.201800070 (2018).
8. Moens, T. G. *et al.* C9orf72 arginine-rich dipeptide proteins interact with ribosomal proteins in vivo to induce a toxic translational arrest that is rescued by eIF1A. *Acta Neuropathol* **137**, 487-500, doi:10.1007/s00401-018-1946-4 (2019).
9. Kanekura, K. *et al.* Poly-dipeptides encoded by the C9ORF72 repeats block global protein translation. *Hum Mol Genet* **25**, 1803-1813, doi:10.1093/hmg/ddw052 (2016).
10. Choi, S. Y. *et al.* C9ORF72-ALS/FTD-associated poly(GR) binds Atp5a1 and compromises mitochondrial function in vivo. *Nat Neurosci* **22**, 851-862, doi:10.1038/s41593-019-0397-0 (2019).

11 Lopez-Gonzalez, R. *et al.* Poly(GR) in C9ORF72-Related ALS/FTD Compromises Mitochondrial Function and Increases Oxidative Stress and DNA Damage in iPSC-Derived Motor Neurons. *Neuron* **92**, 383-391, doi:10.1016/j.neuron.2016.09.015 (2016).
12 Kearse, M. G. *et al.* Ribosome queuing enables non-AUG translation to be resistant to multiple protein synthesis inhibitors. *Genes Dev* **33**, 871-885, doi:10.1101/gad.324715.119 (2019).
13 Tabet, R. *et al.* CUG initiation and frameshifting enable production of dipeptide repeat proteins from ALS/FTD C9ORF72 transcripts. *Nat Commun* **9**, 152, doi:10.1038/s41467-017-02643-5 (2018).
14 Florin, T. *et al.* An antimicrobial peptide that inhibits translation by trapping release factors on the ribosome. *Nature Structural & Molecular Biology* **24**, 752-757, doi:10.1038/nsmb.3439 (2017).
15 Gagnon, M. G. *et al.* Structures of proline-rich peptides bound to the ribosome reveal a common mechanism of protein synthesis inhibition. *Nucleic Acids Research* **44**, 2439-2450, doi:10.1093/nar/gkw018 (2016).
16 Mardirossian, M. *et al.* The Dolphin Proline-Rich Antimicrobial Peptide Tur1A Inhibits Protein Synthesis by Targeting the Bacterial Ribosome. *Cell Chemical Biology* **25**, 530-539.e537, doi:10.1016/j.chembiol.2018.02.004 (2018).
17 Wu, S. *et al.* Diverse roles of assembly factors revealed by structures of late nuclear pre-60S ribosomes. *Nature* **534**, 133-137, doi:10.1038/nature17942 (2016).
18 Greber, B. J. *et al.* Insertion of the Biogenesis Factor Rei1 Probes the Ribosomal Tunnel during 60S Maturation. *Cell* **164**, 91-102, doi:10.1016/j.cell.2015.11.027 (2016).
19 Ma, C. *et al.* Structural snapshot of cytoplasmic pre-60S ribosomal particles bound by Nmd3, Lsg1, Tif6 and Reh1. *Nat Struct Mol Biol* **24**, 214-220, doi:10.1038/nsmb.3364 (2017).
20 Lee, K. H. *et al.* C9orf72 Dipeptide Repeats Impair the Assembly, Dynamics, and Function of Membrane-Less Organelles. *Cell* **167**, 774-788 e717, doi:10.1016/j.cell.2016.10.002 (2016).
21 Tao, Z. *et al.* Nucleolar stress and impaired stress granule formation contribute to C9orf72 RAN translation-induced cytotoxicity. *Human Molecular Genetics* **24**, 2426-2441, doi:10.1093/hmg/ddv005 (2015).
22 Radwan, M. *et al.* Arginine in C9ORF72 dipolypeptides mediates promiscuous proteome binding and multiple modes of toxicity. *Molecular & Cellular Proteomics*, doi:10.1074/mcp.RA119.001888 (2020).
23 Yamada, S. B. *et al.* RPS25 is required for efficient RAN translation of C9orf72 and other neurodegenerative disease-associated nucleotide repeats. *Nat Neurosci* **22**, 1383-1388, doi:10.1038/s41593-019-0455-7 (2019).
24 Mori, K. *et al.* The C9orf72 GGGGCC repeat is translated into aggregating dipeptide-repeat proteins in FTLD/ALS. *Science* **339**, 1335-1338, doi:10.1126/science.1232927 (2013).
25 Green, K. M. *et al.* RAN translation at C9orf72-associated repeat expansions is selectively enhanced by the integrated stress response. *Nat Commun* **8**, 2005, doi:10.1038/s41467-017-02200-0 (2017).

26 Cheng, W. *et al.* C9ORF72 GGGGCC repeat-associated non-AUG translation is upregulated by stress through eIF2alpha phosphorylation. *Nat Commun* **9**, 51, doi:10.1038/s41467-017-02495-z (2018).
