# Supplementary material for: Ribosome inhibition by C9ORF72-ALS/FTD-associated poly-PR and poly-GR proteins revealed by cryo-EM
Source: Nat Commun. 2022 May 19;13:2776. doi: 10.1038/s41467-022-30418-0 (PMC9120013; doi:10.1038/s41467-022-30418-0)
Supplement: Supplementary file 3 — Reporting Summary [file 41467_2022_30418_MOESM3_ESM.pdf]

nature research

Corresponding author(s): Fen-Biao Gao and Andrei A. Korostelev

Last updated by author(s): 3/21/2022

Reporting Summary

Nature Research wishes to improve the reproducibility of the work that we publish. This form provides structure for consistency and transparency in reporting. For further information on Nature Research policies, see our Editorial Policies and the Editorial Policy Checklist.

Statistics

For all statistical analyses, confirm that the following items are present in the figure legend, table legend, main text, or Methods section.

n/a	Confirmed	
☐	☒	The exact sample size (*n*) for each experimental group/condition, given as a discrete number and unit of measurement
☐	☒	A statement on whether measurements were taken from distinct samples or whether the same sample was measured repeatedly
☐	☒	The statistical test(s) used AND whether they are one- or two-sided<br>*Only common tests should be described solely by name; describe more complex techniques in the Methods section.*
☐	☒	A description of all covariates tested
☒	☐	A description of any assumptions or corrections, such as tests of normality and adjustment for multiple comparisons
☐	☒	A full description of the statistical parameters including central tendency (e.g. means) or other basic estimates (e.g. regression coefficient) AND variation (e.g. standard deviation) or associated estimates of uncertainty (e.g. confidence intervals)
☒	☐	For null hypothesis testing, the test statistic (e.g. *F*, *t*, *r*) with confidence intervals, effect sizes, degrees of freedom and *P* value noted<br>*Give P values as exact values whenever suitable.*
☒	☐	For Bayesian analysis, information on the choice of priors and Markov chain Monte Carlo settings
☒	☐	For hierarchical and complex designs, identification of the appropriate level for tests and full reporting of outcomes
☒	☐	Estimates of effect sizes (e.g. Cohen's *d*, Pearson's *r*), indicating how they were calculated

Our web collection on statistics for biologists contains articles on many of the points above.

Software and code

Policy information about availability of computer code

Data collection	Tecan Infinite m1000 pro luminescence reader with Tecan iControl (vs. 2.0.10.0), NanoDrop One, SerialEM (vs. 3.6), IMOD (vs. 4.9.0), GeneMapper (ABI)
Data analysis	Gnuplot (vs. 5.0), Graphpad Prism (vs. 8), cisTEM (version 1.0-beta and pre-release), Frealign v. 9.11 (Aug 2017 release), EMAN (vs.2.07), RSRef(2000), Phenix (1.16-3549), Chimera (vs. 1.7), MacPyMOL: PyMOL v1.7.0.5 enhanced for MacOsX, IMOD (vs. 4.9.0), GeneMarker 3.0.1 (demo version), Coot (vs. 0.8.2; Turtle Bay),

For manuscripts utilizing custom algorithms or software that are central to the research but not yet described in published literature, software must be made available to editors and reviewers. We strongly encourage code deposition in a community repository (e.g. GitHub). See the Nature Research guidelines for submitting code & software for further information.

Data

Policy information about availability of data

All manuscripts must include a data availability statement. This statement should provide the following information, where applicable:
- Accession codes, unique identifiers, or web links for publicly available datasets
- A list of figures that have associated raw data
- A description of any restrictions on data availability

The models generated in this study have been deposited in the RCSB Protein Data Bank under the following accession codes: 7TOO (https://www.rcsb.org/structure/7TOO) (yeast ribosome with GR20), 7TOP (https://www.rcsb.org/structure/7TOP) (yeast ribosome with PR20), 7TOQ (https://www.rcsb.org/structure/7TOQ) (rabbit ribosome with PR20), 7TOR (https://www.rcsb.org/structure/7TOR) (rabbit ribosome with GR20) and 7TOS (https://www.rcsb.org/structure/7TOS) (E. coli ribosome with PR20). The cryo-EM maps used to generate models in this study have been deposited in the Electron Microscopy Database under the following accession codes: EMD-26033 (https://www.ebi.ac.uk/emdb/EMD-26033) (yeast ribosome with GR20), EMD-26034 (https://www.ebi.ac.uk/

emdb/EMD-26034) (yeast ribosome with PR20), EMD-26035 (https://www.ebi.ac.uk/emdb/EMD-26035) (rabbit ribosome with PR20), and EMD-26036 (https://www.ebi.ac.uk/emdb/EMD-26036) (rabbit ribosome with GR20) and EMD-26037 (https://www.ebi.ac.uk/emdb/EMD-26037) (E. coli ribosome with PR20). Source data are provided with this paper.

The coordinate files used in this study are available in the RCSB Protein Data Bank: 1M90 (https://www.rcsb.org/structure/1M90), 1VY5 (https://www.rcsb.org/structure/1YV5), 3JCT (https://www.rcsb.org/structure/3JCT), 3J77 (https://www.rcsb.org/structure/3J77), 4V7U (https://www.rcsb.org/structure/4V7U), 4V88 (https://www.rcsb.org/structure/4V88), 5APN (https://www.rcsb.org/structure/5APN), 5HAU (https://www.rcsb.org/structure/5HAU), 5H4P (https://www.rcsb.org/structure/5H4P), 5LZS (https://www.rcsb.org/structure/5LZS), 5O2R (https://www.rcsb.org/structure/5O2R), 5UYM (https://www.rcsb.org/structure/5UYM), 6HCJ (https://www.rcsb.org/structure/6HCJ), 6FKR (https://www.rcsb.org/structure/6FKR), 6R5Q (https://www.rcsb.org/structure/6R5Q), 6R6Q (https://www.rcsb.org/structure/6R6Q), and 6WDF (https://www.rcsb.org/structure/6WDF). The electron density maps used in this study are available from the Electron Microscopy Database: EMD-1003 (https://www.ebi.ac.uk/emdb/EMD-1003), EMD-4729 (https://www.ebi.ac.uk/emdb/EMD-4729), and EMD-5976 (https://www.ebi.ac.uk/emdb/EMD-5976).

Field-specific reporting

Please select the one below that is the best fit for your research. If you are not sure, read the appropriate sections before making your selection.

[x] Life sciences [ ] Behavioural & social sciences [ ] Ecological, evolutionary & environmental sciences

For a reference copy of the document with all sections, see nature.com/documents/nr-reporting-summary-flat.pdf

Life sciences study design

All studies must disclose on these points even when the disclosure is negative.

Sample size	Cryo-EM Datasets for each complex were collected such that a resolution of ~3 A would be reached. Datasets of >200,000 particles are sufficient by experience on the equipment used. For non-cryo-EM experiments, results were replicated at least once to show reproducibility or multiple times if additional conditions were tested (new antibiotics, new concentration range etc). Duplicate experiments with strong response relative to negative control were deemed sufficient.
Data exclusions	A cryo-EM dataset that yielded low resolution reconstructions (6A) was excluded and the collection was repeated. For in vitro release assay in the presence/absence of erythromycin and polyPR experiments, time points well past saturation of the uninhibited curve were excluded due to 2nd and 3rd order kinetics. For sucrose gradient experiments, if one tube was bumped and the trace was unusable, the experimental condition affected was excluded and but remaining were used unless the positive control was also missing. For toeprinting, if a pellet was lost during precipitation, the condition with all controls was excluded from further analysis. Exclusion criteria were not pre-established.
Replication	For extract and in vitro release experiments, experiments were repeated at least once and up to 8 times and all results were repeatable. Sucrose gradient experiments were replicated 2 or more times and were repeatable. Toe printing experiments from sucrose gradient fractions were replicated twice and both experiments are shown. Cryo-EM experiments were not repeated unless map resolution needed to be improved because this is not usual practice in the field and data collection is very expensive.
Randomization	Randomization was not used for biochemical experiments, instead positive and negative controls were used for each type of experiment. Computational approaches to unbiased particle classification (maximum likelihood classification) include randomizations. Classifications were repeated multiple times typically by varying number of classes or mask position.
Blinding	Blinding was not required because for each sample the structural data were all analyzed using the same methods.

Reporting for specific materials, systems and methods

We require information from authors about some types of materials, experimental systems and methods used in many studies. Here, indicate whether each material, system or method listed is relevant to your study. If you are not sure if a list item applies to your research, read the appropriate section before selecting a response.

Materials & experimental systems

n/a	Involved in the study
[x]	[ ] Antibodies
[x]	[ ] Eukaryotic cell lines
[x]	[ ] Palaeontology and archaeology
[x]	[ ] Animals and other organisms
[x]	[ ] Human research participants
[x]	[ ] Clinical data
[x]	[ ] Dual use research of concern

Methods

n/a	Involved in the study
[x]	[ ] ChIP-seq
[x]	[ ] Flow cytometry
[x]	[ ] MRI-based neuroimaging
